# Assessment of pre and postoperative anxiety in patients undergoing ambulatory oral surgery in primary care

**DOI:** 10.4317/medoral.21929

**Published:** 2017-10-21

**Authors:** Eva Reyes-Gilabert, Luis-Gabriel Luque-Romero, Gracia Bejarano-Avila, Alfonso Garcia-Palma, Angel Rollon-Mayordomo, Pedro Infante-Cossio

**Affiliations:** 1 Odontología, Distrito Aljarafe-Sevilla Norte, Sevilla; 2 Medicina de Familia y Comunitaria, Unidad de Investigación del Distrito Aljarafe-Sevilla Norte, Sevilla; 3 Cirugía Oral y Maxilofacial, Hospital Universitario Virgen Macarena, Sevilla; 4 Departamento de Cirugía, Facultad de Medicina, Universidad de Sevilla

## Abstract

**Background:**

To analyze the pre- and postoperative anxiety level in patients undergoing ambulatory oral surgery (AOS) in a primary healthcare center (PHC).

**Material and Methods:**

Prospective and descriptive clinical study on 45 patients who underwent AOS procedures in the dental clinic of a public PHC of Spain between April and September 2015. Anxiety analysis was carried out with pre- and postoperative anxiety-state (STAI-S), anxiety-trait (STAI-T) and dental anxiety (MDAS) questionnaires. A descriptive, inferential and binary logistic regression analysis were performed for the variables age, sex, educational level, previous experience of oral treatment, type of oral surgery, degree of third molar impaction, surgical time, intraoperative complications, postoperative complications, and pain score with a visual analogue scale (VAS).

**Results:**

The majority were female (57.8%) with a mean age of 33.5+9.6 years. The most frequent procedure was the lower third molar removal (82.2%). The mean pain score on the VAS was 1.6+1.8. The incidence of complications was low (7.8%). There was a statistically significant association between post- and preoperative anxiety (r=0.56, *p*<0.001) and a correlation between pain score and postoperative anxiety (Rho= -0.35, *p*=0.02). The likelihood of postoperative anxiety was related to preoperative anxiety (OR=1.3, *p*=0.03).

**Conclusions:**

AOS in a HPC is safe and should be more encouraged in the public primary care. The emotional impact on users was relatively low, highlighting that the preoperative anxiety levels were higher than the postoperative ones. Psychological factors related to pre- and postoperative anxiety should be considered in the AOS carried out in PC.

** Key words:**Anxiety, oral surgery, ambulatory surgery, primary care, STAI scale, postoperative pain.

## Introduction

Primary care (PC) dentists have gradually expanded their skills and abilities in oral surgery (OS) since demand and hospital´s waiting lists for oral and maxillofacial surgery have increased. In Spain, ambulatory oral surgery (AOS) procedures in PC have been developed in the context of minor surgery programs by general practitioners (1). AOS programs have been already imple-mented in some primary healthcare centers (PHC) of our country as an available option to the user within the health care portfolio, so that only the most complex cases of OS are sent to specialized hospital units. The advantages of AOS lie in the patient’s preference for this type of minor surgery in a closer, familiar and known setting, the reduction of waiting lists, and the significant economic savings avoiding referrals to specialized hospital units (2).

In PC, AOS is proposed for short-term non-complex surgical procedures performed in hard and soft tissues of the oral cavity under local or loco-regional anesthesia, in which no significant complications are expected to occur. The patient is discharged early, without a period of recovery after the anesthetic procedure, being able to go home immediately. The most frequently OS procedure in the public health care portfolio is the surgical removal of third molars, teeth and roots. But also, fenestration of impacted teeth, enucleation of small-sized maxillary cysts, biopsies of hard and soft tissues, surgery of buccal frenulum, and minor surgery of the oral mucosa lesions in which malignant etiology is not suspected, are included (3).

OS usually generate a very high level of anxiety to patients, mainly associated with subsequent postoperative pain and morbidity. It has been linked to injection anesthetic, use of dental handpiece and extraction of teeth (4). This anxiety impacts on the pre- and postoperative clinical care and adherence to treatment in patients who normally have to face several surgical appointments, worsening their health indicators and quality of life (5-7). Various definitions of anxiety have been used for dental procedures (8). In general terms, it can be defined as an individual and subjective experience of multisystemic response to a threatening belief, which constitutes a barrier to the request for oral care (9). Anxiety is characterized by an emotional reaction to a subsequent aversive experience which appears as stress, apprehension, nervousness and worry, with a feeling of self-control loss, and activation of the autonomic nervous system (10).

In the literature, several descriptive studies have been reported highlighting the importance of quantitative measurement of anxiety in different dental procedures using the visual analogue scale (VAS) for pain and the State-Trait Anxiety Inventory (STAI) (11,12). The STAI questionnaire comprises two separate scales that measure two independent concepts: the anxiety-trait (STAI-T) and the anxiety-state (STAI-S). The anxiety-trait is a stable tendency of anxiety of an individual against any situation perceived as a threat. On the contrary, anxiety-state is temporary, and is described as subjective stress, apprehension and hyperactivity of the autonomic nervous system, variable in time and intensity. In our country, dental anxiety has been studied for third molar removal and dental implant placement in university settings and private health care (13,14). However, to our knowledge, there are no studies on the level of pre- and postoperative anxiety in the AOS carried out in a public PHC. Although OS procedures show a relatively short period of postoperative recovery with a low rate of complications, the physical and psychological impact on patients can makes them a stressful experience in PC. Our hypothesis was that OS can provoke patients´ anxiety in the PC setting, and its study can help implement measures to decrease it and improve the health care of the PHC user.

The aim of this article is to assess the anxiety of patients undergoing AOS by the PC dentist, to compare the anxiety-trait with the anxiety-state, to analyze if there is a change in the level of pre- and postoperative anxiety, and to describe the factors that can influence patients’ anxiety.

## Material and Methods

A longitudinal prospective study was conducted in the peri-urban Aljarafe-Sevilla North Sanitary District, Spain. The target population was patients belonging to this district, which in 2015 corresponded to 635,319 people. This population was assisted by 17 PC dentists. The study population was all users attended between April and September 2015 in the dental clinic of the PHC. The study was approved by the bioethics committee of the Virgen Macarena University Hospital and Virgen del Rocio University Hospital of Seville, Spain.

The program of AOS was carried out by a PC dentist and an assistant. Preoperative evaluation and surgical procedure were performed as a day case surgery. An average of 3 patients per day were operated on, apart from those patients attended for routine dental procedures. For diagnosis, an orthopantomography was requested according to the procedure. It was performed in an adequately equipped dental clinic of the PHC. Postoperative follow-up was done in the same setting by the same staff in the first week.

**Figure 1:**
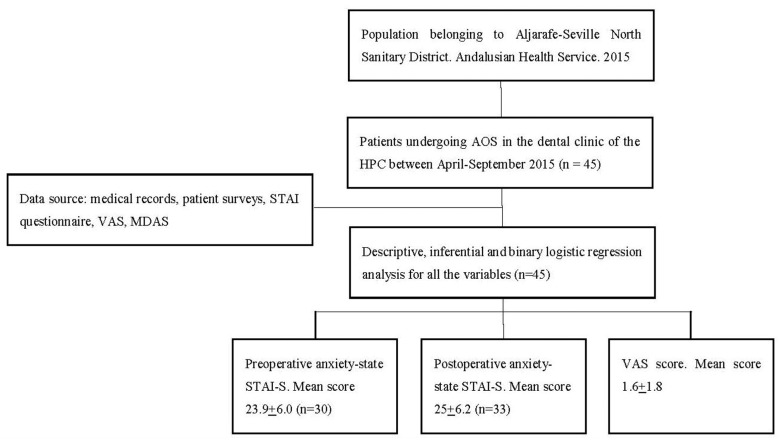
General outline of the study.

- Inclusion criteria. Patients between 18 and 65 years of age, with indication of an AOS procedure under local or loco-regional anesthesia, who accepted and signed informed consent. Patients who were unable to complete the study questionnaires due to a mental or cognitive disorder, treated with anxiolytics, or who had allergies to local anesthetics, infections or pathology that prevented a safe surgical procedure, were excluded.

- Measurement instruments. To quantify the level of anxiety, the anxiety-trait and anxiety-state STAI questionnaires and the modified Corah dental anxiety scale (MDAS), were used. The STAI has been validated and translated into Spanish with reference data available in Spanish patients for OS (11). The anxiety-trait questionnaire has 20 self-assessment questions about habitual situations that the patient perceives as threatening, ranging from 0 to 3, from “almost never” to “almost always.” The anxiety-state questionnaire has 20 self-assessment questions, ranging from 0 to 3, and evaluates the transient emotional state on subjective feelings of stress and apprehension that tend to fluctuate in intensity over time. The total score of the questionnaire ranges from 0 to 60. An anxiety level above 20 is considered for young males and 22 for young females (qualitative variable, percentile-50 in validation studies) (14). The MDAS is a questionnaire specifically designed to measure anticipatory fear and dental anxiety. It consists of 5 multiple-choice questions with scores ranging from 5 (no anxiety) to 25 (maximum anxiety). An anxiety score above 13 points is classified as having high anxiety (qualitative variable) (15).

- Type of procedures. Surgical removal of roots, teeth and impacted and partially impacted third molars, using a muco-periosteal flap, osteotomy, odonto-section and removal with forceps or elevators, depending on the case. Excisional-biopsy of oral mucosa lesions followed by a histological study. At the end of the surgical intervention, postoperative instructions of a hygienic and dietetic type were given and, depending on the case, treatment with analgesics and/or anti-inflammatory drugs and antibiotic therapy was prescribed according to the characteristics of the patient and procedure.

- Data collection. 30 minutes before surgery, while the patient stood in the waiting room, the STAI-S questionnaire and MDAS were given for preoperative assessment. After the surgical intervention, the STAI-S questionnaire and VAS were administered for postoperative assessment. The STAI-T questionnaire was given to be completed later at home without probable stress or anxiety and to be returned at the first-week follow-up visit. The general outline of the study is depicted in Figure 1.

- Study variables. Dependent variables: pre- and postoperative anxiety-state with the STAI-S questionnaire. Independent variables: age, sex, educational level, previous experience of oral treatment, type of oral surgery, degree of third molar impaction, surgical time, intraoperative complications, postoperative complications, pain score (VAS), anxiety-trait with the STAI-T questionnaire, and MDAS.

- Statistical procedures. For descriptive purposes, the study of qualitative variables was presented by the absolute and relative frequencies, and quantitative variables by the mean and standard deviation (SD). A bivariate analysis of the data was performed. In order to compare qualitative variables, contingency tables were created and Chi square test or Fisher’s modification were used (in sparsely populated tables or for expected frequencies less than 5). To determine the association between dichotomous and quantitative variables, the Student t test were used once the symmetry of the distribution was determined using the Shapiro-Wilk test. Student’s t test for repeated measures was used to compare pre- and postoperative changes in anxiety-state level. Correlation and binary logistic regression analyzes were used to determine the relationship between variables. The values were expressed together with 95% confidence interval. The hypothesis contrasts were done in a bilateral manner, setting the confidence level at 95%. The values of *p*<0.05 were considered significant. The collected data were analyzed in SPSS for Windows v.18 (SPSS Inc. USA).

## Results

Data on age, sex, educational level and experience of previous oral treatment, as well as AOS procedures and complications, are shown in  Table 1. Forty-five surgical procedures were performed, of which 37 (82.2%) were third molar removals. The average surgical duration was 9.7±4.5 minutes (maximum 20, minimum 4). The incidence of intraoperative complications was low (7.8%), while there were no postoperative complications. The histological diagnosis of the excised tumors corresponded to oral papillomas in all cases.

**Table 1:**
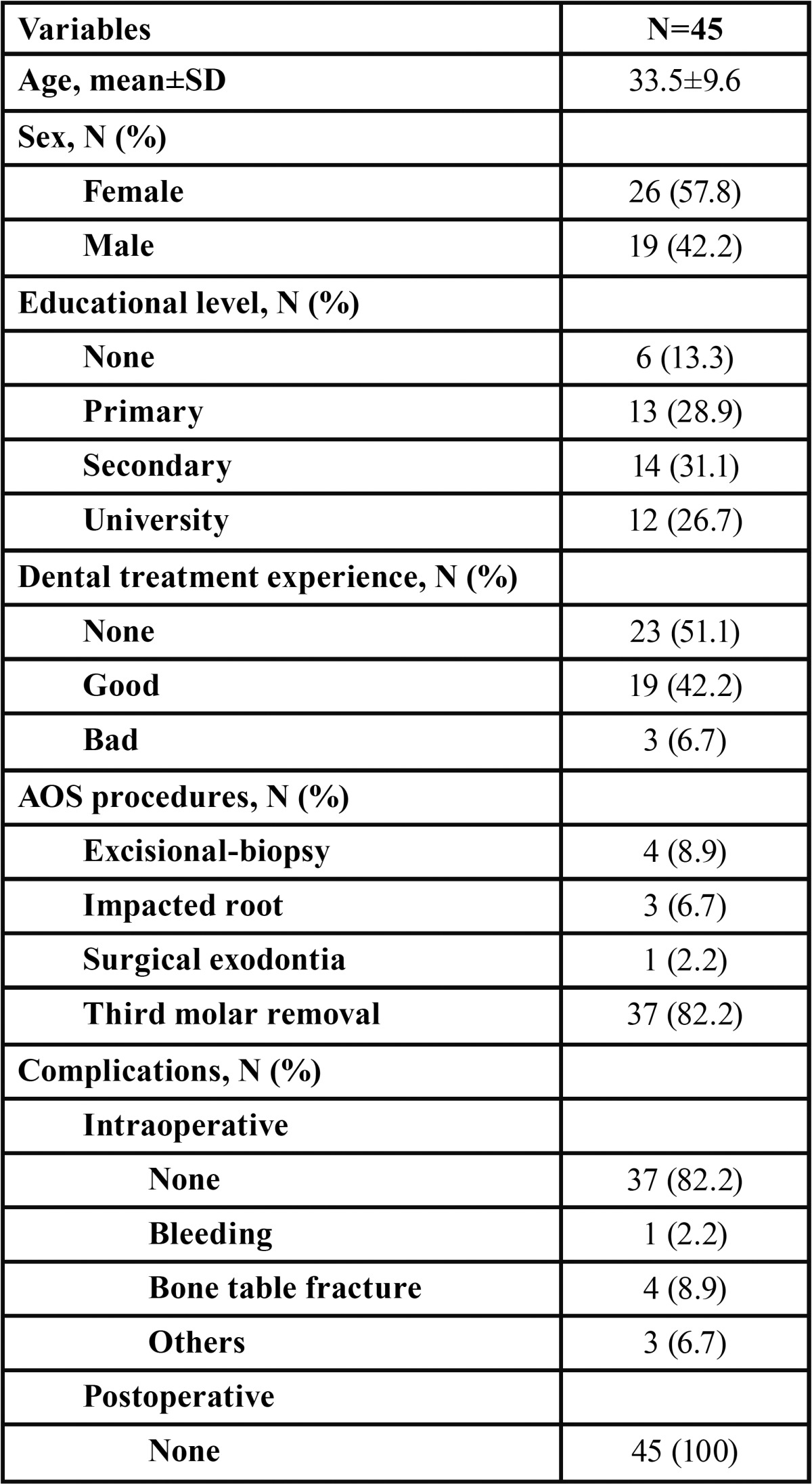
Characteristics of the patients, procedures performed and complications (n = 45).

The anxiety-trait, pre-and postoperative anxiety-state and pain scores, are shown in  Table 2. The distribution of explanatory variables with respect to pre- and postoperative anxiety-state are shown in  Table 3 and  Table 4. The preoperative STAI-S questionnaire registered 30 patients (66.7%) with anxiety scores (total score=23.9±6), being 23.8±5.2 for males and 24±6.6 for females (*p*=0.93). Postoperative anxiety with the STAI-S scale was achieved by 33 patients (73.3%), with a mean of 25±6.2, being 24.7±6.4 for males and 25.3±6.1 for females (*p*=0.74). Eighteen patients (40%) had severe anxiety-phobia with the MDAS questionnaire. The mean score on the VAS was 1.6±1.8 (minimum=0, maximum=6) and in 33.3% the pain score was zero. Eighty percent of patients reported pain intensity less than or equal to 2. A statistically significant correlation was found between post- and preoperative anxiety (r=0.56, *p*<0.001) and a correlation between pain score and postoperative anxiety (Rho= -0.35, *p*=0.02). A statistically significant association between pre- and postoperative anxiety and the degree of third molar impaction were found, with a pre- and postoperative anxiety mean of 24.0±5.4 and 20.8±6.8, respectively.

**Table 2:**
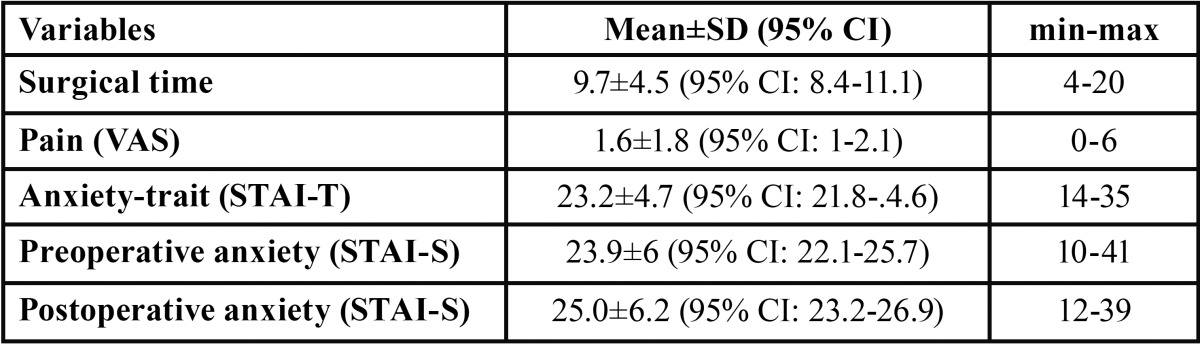
Anxiety-trait, pre-and postoperative anxiety and pain scores.

**Table 3:**
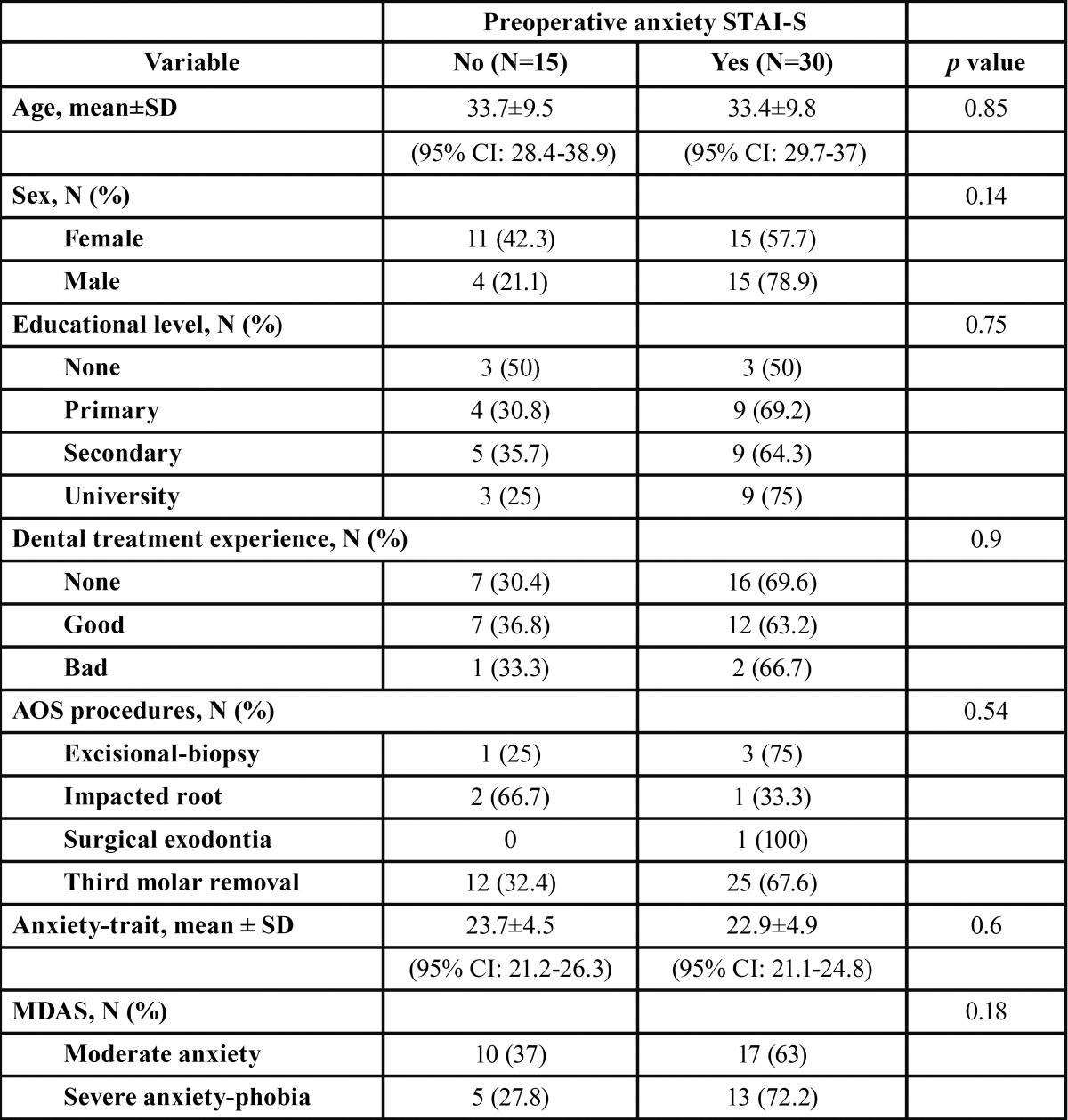
Distribution according to the presence of preoperative anxiety (STAI-S).

**Table 4:**
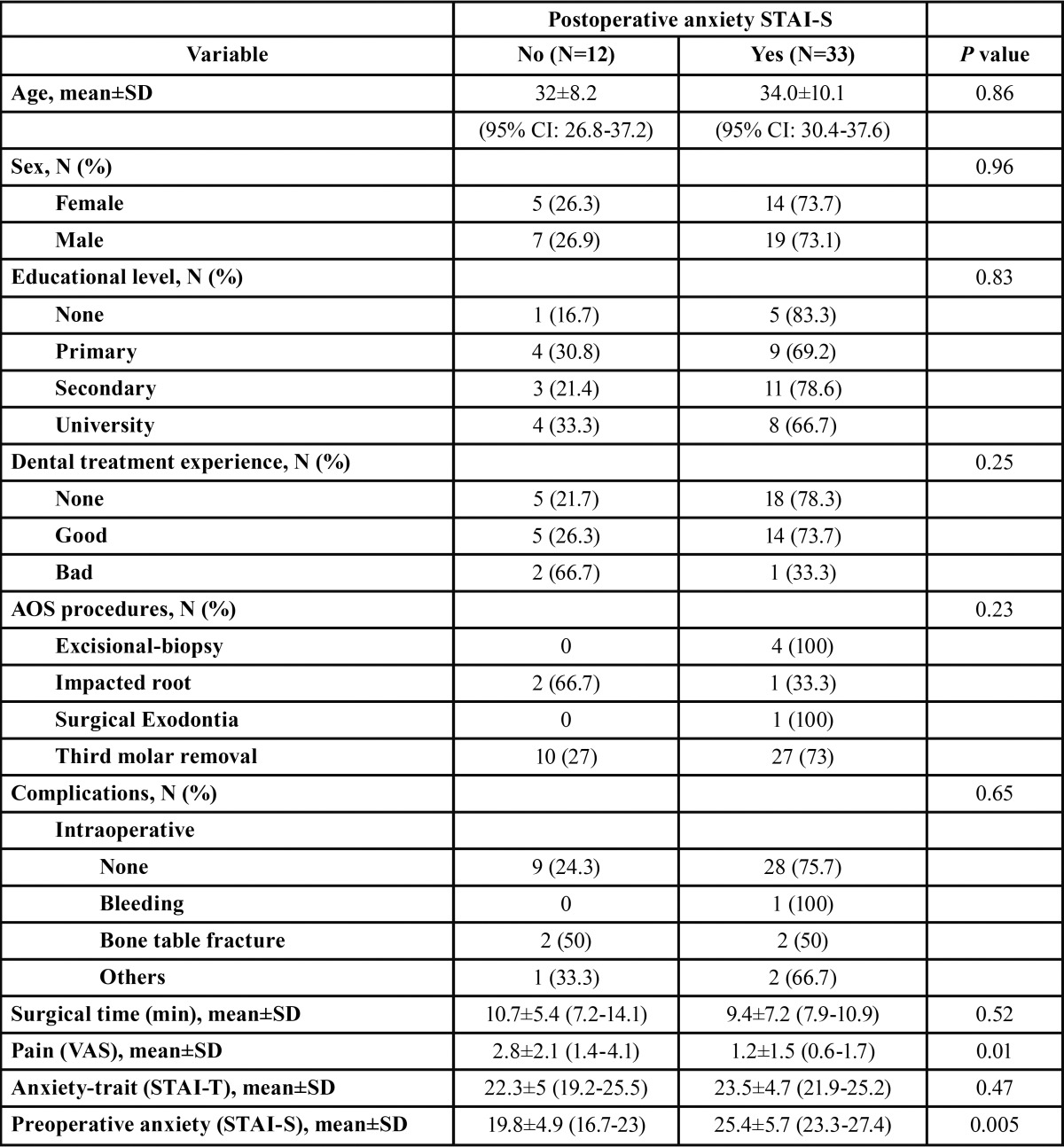
Distribution according to the presence of postoperative anxiety (STAI-S).

The statistical likelihood of having postoperative anxiety was related to preoperative anxiety (OR=1.3, 95% CI=1.03-1.59) (*p*=0.03) by means of binary logistic regression analysis controlling for age, sex, educational level, previous experience of oral treatment, surgical time, and intraoperative complications.

## Discussion

Minor surgery carried out by general practitioners in the PC setting of Spain is safe and satisfactory for the user, as well as effective and cost-effective (16,17). However, although the number of PC dentist who practice OS is increasing, AOS programs are less developed in public PHCs. For their implementation, it is essential to make a correct selection of cases, accomplish an adequate organizational scheme in the PHC, and promote the motivation and change of the mentality of dentists (18). In this context, this study of pre- and postoperative anxiety levels in PC has emerged in order to promote better clinical practice guidelines and improve care for PHC users.

The main concern of the patient undergoing an OS procedure is to minimize the experience of pain and its consequences such as bleeding, inflammation, and disorders in activities of daily living. Fear and anxiety can negatively influence postoperative pain and patient recovery, and thus, to achieve a reduction in the effects of anxiety can lead to more satisfactory procedures (20). This study intended to identify changes in patient´s anxiety using self-administered questionnaires before and after OS procedures performed in the dental clinic of a peri-urban PHC of our country. For this purpose, we used the STAI questionnaire which was widely accepted and validated into the Spanish language and showed solid results in previous epidemiological studies (12,14,19).

The lower third molar removal was the most frequent AOS procedure in our PHC, in 82.2% of patients. The results of our study showed that, to a greater degree of dental impaction and, consequently, to a greater difficulty of the surgical procedure, the patients presented greater preoperative anxiety, showing a significant association between the pre- and postoperative anxiety.

Pre- and postoperative anxiety on patients undergoing AOS was not significantly related to sex in our study. Lago-Méndez *et al.* (21) reported higher dental anxiety in females than in males, and Hakeberg *et al.* (22) found higher levels of anxiety-trait in females, although others have not reported such differences (23,24). Other studies have found a higher level of anxiety in younger people or less educational level (8,24). In our setting, neither age nor level of education was relevant. Previous experience of oral treatment as a confounding variable indicated that most patients had no prior experience or had no experience. The patients did not reflect intense pain in relation to the procedure according to the VAS. Intraoperative complications were few and of little consideration and, therefore, not relevant in anxiety levels.

In our study, there was a low percentile of anxiety-trait. The percentile in the pre- and postoperative anxiety-state increased after the AOS procedures. A statistical association between anxiety-trait and preoperative anxiety-state was found but not with post-operative anxiety-state, indicating that the most anxious patients were those with greater preoperative anxiety. A relationship between the difficulty of the third molar removal and the pre-and postoperative anxiety state was also distinguished (25).

There was no statistical correlation between the surgical time and the pre-and postoperative anxiety-state, nor with the VAS, although as the surgical time increased, the VAS score increased. The MDAS provided an overview of dental anxiety, although it seems unspecific for OS. The comparison of the MDAS results with other previous studies that measured dental anxiety showed that many patients had severe anxiety/phobia at the dental office (14).

This prospective study posed several limitations. First, the small size of the sample, being a single-center study. Second, any study lacking a control group has drawbacks, and its results should be interpreted with caution. Third, the follow-up period was short and limited to the anxiety before and 1-week after AOS procedure, without considering that the mid-term outcome could be altered if complications arose. Finally, the level of anxiety of patients may be influenced by many variables such as the information given to the patient prior to the procedure (26), or the use of drug premedication with anxiolytics or sedation, which in our patients were not administered. In the future, new studies could be designed to specifically study drug medication for anxiety, and improvements to clinical practice guidelines for PHC users could be implemented.

## Conclusions

The findings of the present study showed that AOS procedures in a PHC are safe, with few complications, and should be more encouraged in public PC. The emotional impact on users was relatively low, either because of the good functioning of the PHC or because there were no relevant postoperative complications. The preoperative anxiety levels were higher than the postoperative ones. Psychological factors related to pre- and postoperative anxiety should be considered in the AOS carried out in PC.
